# Prioritization of ethical concerns regarding HIV molecular epidemiology by public health practitioners and researchers

**DOI:** 10.1186/s12889-024-18881-4

**Published:** 2024-05-29

**Authors:** Anne L. R. Schuster, Juli Bollinger, Gail Geller, Susan J. Little, Sanjay R. Mehta, Travis Sanchez, Jeremy Sugarman, John F. P. Bridges

**Affiliations:** 1 Department of Biomedical Informatics, The Ohio State University College of Medicine, 1800 Cannon Drive, Columbus, OH 43016 USA; 2 Berman Institute of Bioethics, Johns Hopkins University, Baltimore, MD USA; 3 School of Medicine, Johns Hopkins University, Baltimore, MD USA; 4 Division of Infectious Disease, University of California San Diego, San Diego, CA USA; 5 Department of Epidemiology, Rollins School of Public Health, Emory University, Atlanta, GA USA; 6 Department of Health Behavior and Society, Bloomberg School of Public Health, Johns Hopkins University, Baltimore, MD USA

**Keywords:** HIV surveillance, Molecular epidemiology, Public health surveillance, Best–worst scaling, Ethics

## Abstract

**Background:**

HIV molecular epidemiology (HIV ME) can support the early detection of emerging clusters of new HIV infections by combining HIV sequence data routinely obtained during the clinical treatment of people living with HIV with behavioral, geographic, and sociodemographic information. While information about emerging clusters promises to facilitate HIV prevention and treatment efforts, the use of this data also raises several ethical concerns. We sought to assess how those working on the frontlines of HIV ME, specifically public health practitioners (PHPs) and researchers, prioritized these issues.

**Methods:**

Ethical issues were identified through literature review, qualitative in-depth interviews, and stakeholder engagement. PHPs and researchers using HIV ME prioritized the issues using best–worst scaling (BWS). A balanced incomplete block design was used to generate 11 choice tasks each consisting of a sub-set of 5 ethical concerns. In each task, respondents were asked to assess the most and least concerning issue. Data were analyzed using conditional logit, with a Swait-Louviere test of poolability. Latent class analysis was then used to explore preference heterogeneity.

**Results:**

In total, 57 respondents completed the BWS experiment May–June 2023 with the Swait-Louviere test indicating that researchers and PHPs could be pooled (*p* = 0.512). Latent class analysis identified two classes, those highlighting “Harms” (*n* = 29) (prioritizing concerns about potential risk of legal prosecution, individual harm, and group stigma) and those highlighting “Utility” (*n* = 28) (prioritizing concerns about limited evidence, resource allocation, non-disclosure of data use for HIV ME, and the potential to infer the directionality of HIV transmission). There were no differences in the characteristics of members across classes.

**Conclusions:**

The ethical issues of HIV ME vary in importance among stakeholders, reflecting different perspectives on the potential impact and usefulness of the data. Knowing these differences exist can directly inform the focus of future deliberations about the policies and practices of HIV ME in the United States.

**Supplementary Information:**

The online version contains supplementary material available at 10.1186/s12889-024-18881-4.

## Background

HIV ME (HIV ME) is currently used in both research and public health activities related to HIV prevention, treatment, and surveillance in the United States. Molecular HIV surveillance (MHS) promises to facilitate the early detection of HIV transmission clusters, thereby allowing public health practitioners (PHPs) to implement targeted interventions. HIV ME has also been used to describe emerging epidemics [1, 2], drug-resistance dynamics [3–7], and to predict risk factors associated with transmission [8–10]. Furthermore, MHS is a key component in the Ending the HIV Epidemic in the U.S. (E.H.E.) initiative, which aims to reduce new HIV infections by 90% by 2030 [11, 12]. In the United States, MHS relies upon partial HIV gene sequence data obtained through HIV drug resistance testing, which is a common component of routine clinical care of individuals living with HIV.

While the U.S. Centers for Disease Control and Prevention (CDC) has stated that MHS has assisted in the detection of hundreds of growing transmission clusters across the country [13], its use in HIV surveillance has raised social and ethical concerns related to consent, privacy and confidentiality leading some to call for a moratorium on its use in public health [14, 15]. Some critics fear that MHS data may intersect with laws that criminalize HIV transmission or non-disclosure, while others express concern that identification and public disclosure of HIV transmission clusters may lead to increased risk of stigma and discrimination in affected communities [16–24]. In 2023, the Presidential Advisory Council on HIV/AIDS (PACHA) passed a resolution calling on public health agencies and others to take a variety of actions in response to such concerns [15]. These include meaningfully engaging with communities regarding MHS, gathering evidence on its use, obtaining consent from individuals for use of their HIV viral sequence data in public health, and addressing HIV criminalization laws [15].

Evidence is needed on the direct benefits of using HIV ME for public health purposes, including on interrupting or preventing HIV transmission, on intermediate outcomes (e.g., HIV testing, pre-exposure prophylaxis uptake, and viral suppression), and on outcomes for vulnerable populations [19, 24, 25]. The lack of evidence makes it challenging for stakeholders to weigh the benefits and risks of the practice. Moreover, no systematic evaluation has been done on how different stakeholders, such as researchers, public health practitioners (PHPs), persons living with HIV and persons living without HIV at increased risk of acquiring it, perceive the use of HIV ME and its implications. To begin to address this knowledge gap, one study explored the views and attitudes of persons living with HIV and persons living without HIV at risk of acquiring it towards HIV ME and recommended further systematic data collection from key stakeholders to inform policymaking and practice in HIV ME [26].

We sought to assess how PHPs and researchers using HIV ME in the United States prioritize the associated ethical issues. There is value in understanding how stakeholders prioritize ethical concerns as they can and should promote a more informed discussion between researchers, PHPs, and other stakeholders about the future policies and practices on the appropriate uses of HIV ME. Similarly, there is value in understanding how different methods such as emerging choice experiment methods can be used to assess ethical concerns and to study differences among various types of professionals using molecular epidemiology.

## Methods

### Methods of identification

The identification of ethical issues surrounding the use of HIV ME in public health and research was informed by the peer-reviewed and grey literature, engagement with HIV ME experts in in public health and research, and the results of earlier qualitative interviews with persons living with HIV and persons living without HIV at increased risk of acquiring it [26]. Six expert stakeholders were selected based on their leadership, and active roles in publishing and providing guidance on the methods, application, and ethical considerations related to using HIV ME in both research and public health contexts. These stakeholders provided input on the relevant ethical issues to be considered in our study. This process resulted in a list and brief description of 11 candidate ethical issues to include in the BWS experiment. Table 1 presents the final list of objects included in the BWS experiment.

**Table 1:** Ethical issues in BWS experiment

Ethical issue	Description	Ref^a^
Limited evidence of benefits	HIV ME results may be used to inform HIV prevention and treatment strategies and reduce HIV transmissions. The evidence may be limited regarding the benefits of using HIV ME. Evidence from controlled trials or prospective empirical observations could further establish its benefits.	[19, 24, 25]
Lack of individual consent	Obtaining informed consent involves providing an individual with comprehensive information about HIV ME, including its purpose, risks, benefits, and uses of data, and then obtaining the individual’s voluntary agreement to participate. Some programs may not be required to obtain individual consent for the use of data in HIV ME, such as for HIV surveillance or research involving de-identified data.	[27–29]
Lack of directly disclosing data use	Disclosure refers to informing individuals that their personal information, such as their HIV genetic sequences, will be used by programs for HIV ME prior to collecting it. Programs that use HIV ME do not routinely disclose the use of an individual’s information prior to it being collected.	[15]
Lack of an opt-out option	An opt-out option refers to a process where individuals can withdraw their data from use after it has been collected. Programs that use HIV ME do not routinely give individuals an opt-out option.	[29]
Limited resources for other activities	HIV ME may be used to prioritize public health resources for activities expected to have a greater impact on increasing case detection and interrupting HIV transmission. However, it is unclear how the use of resources for HIV ME impacts the allocation of resources for other standard HIV public health activities.	[15]
Increase risk of legal prosecution	In many jurisdictions of the United States, HIV transmission and nondisclosure of HIV status are criminal offenses. If results from HIV ME could be used to infer close linkage between individuals, that might increase the risk of individuals being legally prosecuted.	[22, 27]
Re-use of data collected for clinical purposes	Some programs systematically re-use data collected during routine HIV clinical care for HIV ME. The re-use processes may not promote shared decision making about data usage between persons living with HIV and their clinician. They may also not promote trust between persons living with HIV, their clinician, and the programs re-using the data.	[16]
Infer source of HIV transmission	The use of HIV ME may enable programs to identify persons they believe to be the source of a particular infection. This can be a concern though because there is always uncertainty in the results that may be lost, when reporting or acting on the results.	[19]
Infer directionality of HIV transmission	The use of HIV ME may enable programs to infer the directionality of HIV transmission from one person to another. The ability to infer the directionality of HIV transmission depends on different factors including the methods used to generate the datasets. There are questions about the value of being able to infer the directionality of HIV transmission.	[16]
Increase risk of harm towards individuals	Results from programs using HIV ME can potentially reveal characteristics about individuals that increase their risk of experiencing discrimination. Discrimination is often a consequence of stigma and can occur when unfair actions are taken against individuals based on their belonging to a stigmatized group.	[17, 19, 20]
Increase risk of stigma towards groups	Results from programs using HIV ME could lead to increased stigma towards communities over-represented in the HIV epidemic. Communities disproportionately affected by HIV are also often affected by stigma associated with, among other things, gender identity, sexual behavior, or use of injection drugs.	[16, 17, 20]

^a^*Ref* reference

### Method for prioritization

Best–worst scaling (BWS) was used to prioritize the ethical issues [30]. BWS uses an experimental design to generate choice tasks comprised of subsets of objects (i.e., ethical issues) [31, 32]. Then respondents assess what is the best and worst object in a choice set. BWS experiments have advantages over other prioritization methods such as Likert scales [32–34] and was chosen because of its simplicity, low respondent burden, and strengths in measuring priorities [35, 36]. BWS is increasingly used to assess the priorities of a wide array of stakeholders in medicine [37] and in other fields [38].

### Survey instrument

The survey instrument followed good research practices [39] and began with an introductory section that provided relevant contextual information. The introduction informed participants of the study’s purpose, that their participation was voluntary, and that their individual responses would be kept confidential. The introduction also provided a definition of HIV ME and its uses in research or public health for HIV prevention, treatment, or surveillance in the United States. The next section of the survey collected information about participants, including about their field of work, number of years working in their field, and institutional location.

**Fig. 1:**
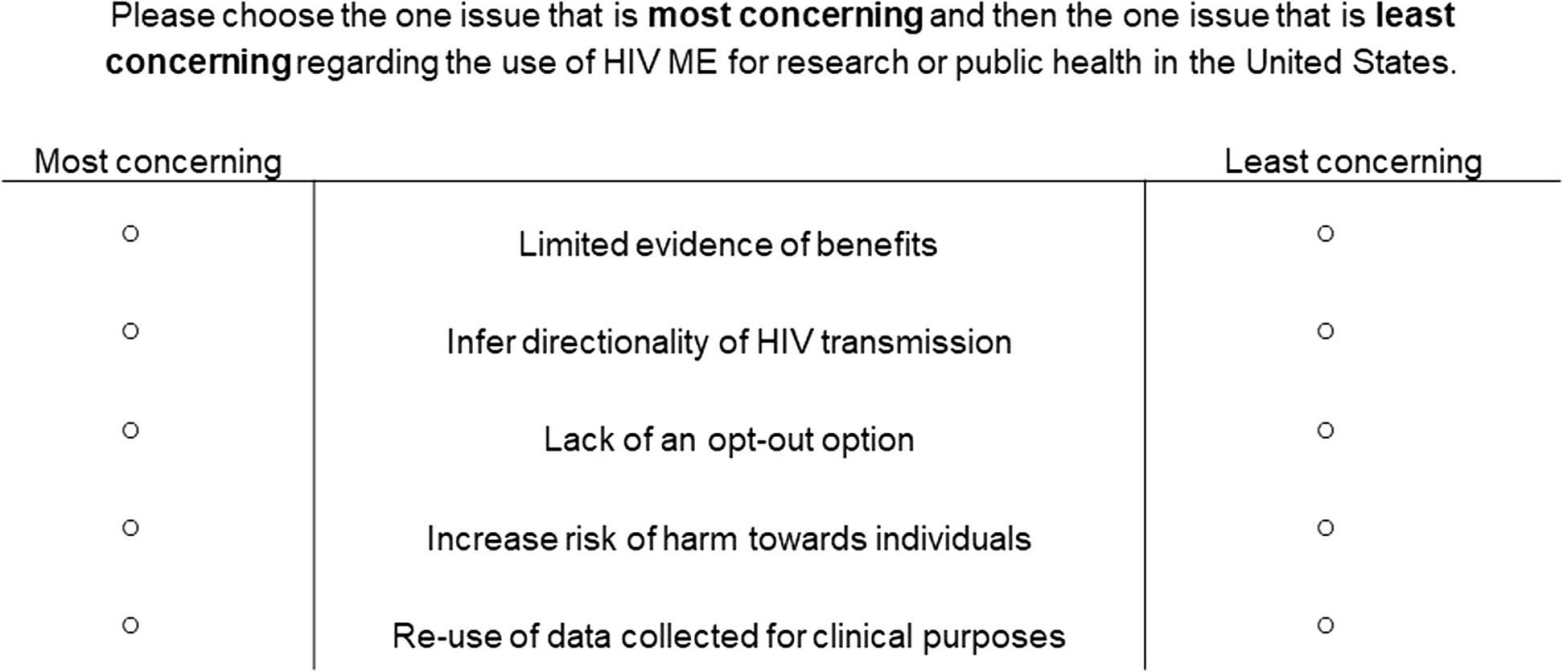
Example BWS choice task

The BWS instrument within the survey was developed specifically for this study and included two parts (Supplementary material). The first was an orientation exercise used to explain each ethical issue and to ask about which issues were a relevant concern (yes/no). The purpose of the orientation exercise was to encourage respondents to read the descriptions for each issue and not to provide data for analysis. The second part elicited priorities of the ethical issues using a series of 11 BWS choice tasks, each of which consisted of a sub-set of 5 ethical issues. All the ethical issues were phrased in the negative to avoid response bias due to directionality of phrasing. The underlying latent, subjective continuum was degree of concern, where each choice set was introduced by the statement: “Please choose the most concerning and then the least concerning ethical issue when using HIV ME for research or public health in the United States.” The choice tasks were introduced using an example task (Fig. 1). A balanced incomplete block design was used to generate the 11 choice tasks where each ethical issue occurred and co-occurred with other issues the same number of times.

### Survey development

The survey development process included pre-testing and piloting to assess the instrument’s burden as well as to ensure content validity and relevance to potential respondents [39]. The expert stakeholders were further engaged in virtual pre-testing interviews that included the think-aloud technique and lasted an average of 60 min. Stakeholders reviewed the survey overall and the descriptions of the ethical issues specifically. Interviewers probed the salience of each ethical issue as well as the accuracy and clarity of their descriptions during pretesting. Pre-testers were asked about the amount of time participants would need to complete the survey and whether other information should be collected about the respondents or whether other ethical issues should be considered. The survey instrument was revised based on pre-testers’ insights and perspectives, including revisions to the labels and descriptions of ethical issues.

A pilot survey was then designed and programmed in Qualtrics (Qualtrics, Provo, Utah). Ten individuals from the study team and the expert stakeholder group piloted the survey to assess its functionality and feasibility. Based on qualitative feedback and quantitative analysis of results from the pilot, the survey was revised to improve its functionality on mobile devices and to improve the clarity of two descriptions. The final survey was programmed in Qualtrics.

### Fielding the survey

We targeted respondents who conduct HIV ME research, and PHPs from U.S. public health departments who are: (1) leaders with oversight of HIV cluster detection and response programs that include molecular cluster detection, and (2) staff members responsible for implementing HIV molecular cluster detection and/or response.

Potential respondents were identified by their: (1) authorship of peer reviewed publications; (2) presentations at major HIV conferences; (3) leadership roles in national societies/centers; (4) publicly available lists of HIV-focused public health officials (e.g., lists from the National Alliance of State and Territorial AIDS Directors; and through (5) the project’s Expert Advisory Board members.

Potential respondents were invited to opt-in to participate via email from the project team directly or on behalf of the team through members of its Expert Advisory Board. The invitation to participate described the survey and its purpose as well as how the results would be used to inform future deliberations about the policies and practices of HIV ME. Potential respondents could opt-in to participate by providing their email address via a brief online survey. Individuals who opted-in to participate in the study were subsequently sent an individual online survey link administered via Qualtrics. The survey was fielded from May 16, 2023 to June 28, 2023. Respondents were not compensated for participating.

### Data analysis

BWS scores were calculated by subtracting the number of times an object was selected as least concerning from the number of times it was selected as most concerning and then dividing each count by the total number of times the object appeared during the experiment. Conditional logistic analysis was conducted using a sequential best–worst choice assumption and stratified by researcher versus PHP. Effects coding was used for the conditional logit analysis. Importance scores were then calculated by rescaling coefficients from the conditional logit to a probability ratio scale that ranges from 0 to 100 [40]. The rescaled importance scores follow ratio scaling where, for example, an item with a score of 10 can be interpreted as being twice as concerning as an item with a score of 5. A Swait-Louviere test was used to test for the poolability of respondents [41].

Preference heterogeneity was assessed using latent class analysis [42], an approach that is increasingly used [43]. We used latent class conditional logit models to identify different groups or “classes” of individuals, with each class having distinct preferences. This involves estimating parameters that determine which class a given individual is likely to be a member of [44]. For each class, a separate conditional logit model is estimated. Wald tests were used to test differences in specific coefficients between the classes. We also used chi-square tests to examine the association between class membership and respondent characteristics. A key aspect of this exploration included assessing whether the location of participants’ institutions, specifically in states with HIV or sexually transmitted infection (STI) criminalization laws, had an association with class membership. To facilitate this, we sourced data from the U.S. CDC regarding state-specific criminalization laws related to HIV and STIs [45]. Next participants were categorized into two groups: (i) those in states without specific criminalization laws; and (ii) those in states with laws that either criminalize STIs or infectious diseases that might include HIV, or specifically target HIV exposure or actions that could potentially expose another person to HIV [45]. All data analysis was conducted with Stata SE version 17 (StataCorp LLC).

### Oversight

The expert stakeholder engagement that guided survey development was deemed non-human subjects research by the Johns Hopkins School of Medicine (JHM #IRB IRB00354016). The survey was deemed non-human subject research by the Ohio State University College of Medicine Institutional Review Board (OSU IRB #2022E1207). As such, explicit informed consent was not required. However, the survey introduction provided information about the nature of the survey questions, the expected time required to answer them, that participation was voluntary, that individual responses would be treated confidentially, and the survey could be stopped at any time. Next, was a statement: “If you are willing to participate, please click the ‘Next’ button at the bottom of this page.” An advisory board also provides oversight to the project overall.

## Results

In total, 90 professionals opted-in to receive the survey; 57 completed it, including 29 researchers and 28 PHPs. The respondents differed in the number of years they reported working in their respective field; one half (50.0%) of PHPs had worked in their field more than 10 years while over two-thirds (69%) of researchers had worked in their field for over 10 years. All researchers had experience with HIV ME in the U.S. and most (60.7%) conducted research with a focus solely on the U.S. setting. PHPs represented 22 public health jurisdictions from across the United States.

The results of the Swait-Louviere test failed to reject the null hypothesis (*p* = 0.512) that the estimated parameters between researchers and PHPs were the same. This indicated that the data from these two groups could be pooled. In the pooled analysis, respondents prioritized legal prosecution, group stigma, and individual harm the most, and limited resources, data re-use, and lack of an opt-out option the least (Table 2).

**Table 2:** BWS scores and aggregate conditional logit model

	**BWS score**^**a**^	**Aggregate model**^**b**^	**Stratified model importance scores**^**b**^	
		**Importance score (*n* = 57)**	**Researchers (*n* = 29)**	**Public health (*n* = 28)**
Risk of legal prosecution	0.33	16.48	15.79	9.35
Group stigma	0.31	15.78	18.81	23.58
Individual harm	0.27	14.69	13.27	12.49
Infer source	0.04	9.34	11.98	14.45
Lack of disclosure	0.00	8.23	6.39	7.17
Limited evidence	-0.01	7.66	4.38	5.01
Infer directionality	-0.02	7.93	8.17	8.90
Lack of consent	-0.06	7.29	8.18	7.12
Lack of opt-out	-0.15	5.76	6.89	5.83
Data re-use	-0.31	3.85	3.36	4.10
Limited Resources	-0.39	3.00	2.77	2.00
Log likelihood^**c**^	–	-1683.1	-852.4	-826.1

^a^BWS scores calculated by subtracting least-concern count for each object from its most-concern count and dividing by the number of times the object appeared in the survey (5 × N)

^b^Importance scores calculated by rescaling coefficients from conditional logit on a ratio scale from 0 to 100. With ratio scaling an item with a score of 10, for example, can be interpreted as being twice as concerning as an item with a score of 5

^c^Log likelihood from aggregate and stratified models indicate that we fail to reject hypothesis of parameter equality via Swait-Louivere test [41]

**Fig. 2:**
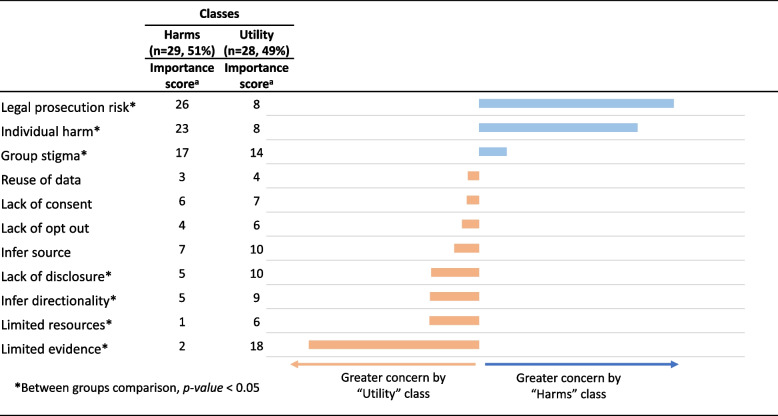
Differences in endorsing ethical issues by latent class membership (2-class model). ^a^Importance scores calculated by rescaling coefficients from conditional logit on a ratio scale from 0 to 100. With ratio scaling an item with a score of 10, for example, can be interpreted as being twice as concerning as an item with a score of 5

Latent class analysis revealed heterogeneity of priorities (as reported by importance scores) clustered in two groups: a “Harms” group (*n* = 29) who prioritized concerns about potential risk of legal prosecution (*p* < 0.001), individual harms (*p* < 0.001), and group stigma (*p* = 0.002); and a “Utility” group (*n* = 28) who prioritized concerns about limited evidence of benefits (*p* < 0.001), uncertain impact on resource allocation (*p* < 0.001), lack of routinely disclosing use of data (*p* = 0.045), and the potential to infer the directionality of HIV transmission (*p* = 0.022). The two groups differed significantly in how they prioritized seven ethical issues (Fig. 2). There were no statistically significant differences in the characteristics of members across classes (Table 3).

**Table 3:** Respondent characteristics by class (*n* = 57)

Characteristic, n (%)	Harms class (*n* = 29, 51%)	Utility class (*n* = 28, 49%)	*p*-value
Public health practitioner	15 (51.7%)	13 (46.4%)	0.69
More than 10 years of experience	18 (62.1%)	16 (57.1%)	0.71
Institution in setting with STI/HIV criminalization laws^a^	19 (65.5%)	20 (71.4%)	0.63

^a^Participants were categorized into states without criminalization laws or those with HIV/STI criminalization laws based on the CDC’s analysis and categorization of state laws [45]

## Discussion

We have explored the ethical issues related to HIV ME by using BWS to elicit the priorities of PHPs and researchers who use HIV ME in their work. Our results revealed two distinct perspectives on the ethical issues: one emphasizing the potential harms to individuals and groups, and the other emphasizing the utility and effectiveness of the approach. These findings relate directly to the increased attention on addressing the ethical issues of using HIV ME in research and public health in the United States. This includes recently revised guidelines from the CDC [46] as well as four proposals presented for federal stakeholders to consider regarding molecular HIV surveillance programs, including recommendations about incorporating opt-outs and plain-language notifications about the use of HIV viral sequence information for public health purposes [29]. Despite these recommendations, other researchers have reported general support and limited concerns about the use of HIV ME in public health and research among persons living with HIV and persons living without HIV at increased risk of acquiring it [26] – evidence that contrasts with the concerns raised by some critics of HIV ME. These findings highlighted the importance of engaging with a diversity of stakeholders, including those who may not have been previously engaged as widely.

Our study contributes to this important and ongoing conversation by presenting the preferences of two groups of professionals not previously engaged systematically. Our results indicated that the preferences of the two groups – researchers and PHPs – were similar enough to be pooled and analyzed using a joint model. The aggregated importance scores showed that the potential added risk of legal prosecution was the most concerning ethical issue and was more than five times as influential as the uncertain impact on the allocation of limited resources, which was the least concerning issue. The prioritization of added risk of legal prosecution is consistent with prior work that has identified this issue as a main ethical concern of using HIV ME [16, 17, 24, 27, 29]. Legal prosecution refers to the risk of using HIV genetic data as evidence in criminal cases against people living with HIV who are accused of transmitting or exposing others to HIV. This practice has been widely criticized for being discriminatory, stigmatizing, and undermining public health efforts to prevent and treat HIV [27]. It was unexpected that the uncertain impact of HIV ME on the allocation of limited resources was the least prioritized issue given that this issue has been discussed at PACHA meetings [15]. One possible explanation is that our participants did not perceive this issue as directly relevant to their work or interests, or that they assumed that HIV ME would not significantly affect the resource allocation decisions.

While we did not expect the preferences of researchers and PHPs to be poolable, there was significant heterogeneity among respondents, where latent class analysis identified two distinct classes of participants. While the classes were not associated with profession or other observable characteristics, the ethical issues prioritized by each class seemed to reflect a coherent and consistent set of values and concerns. The “Harms” class focused on the issues that could cause social harm to individuals or groups who are affected by the actions – inadvertent or not – that result from the use of HIV ME. The Harms class may have a stronger sense of responsibility to the people living with HIV or at risk of HIV infection and may be more sensitive to the potential negative consequences of HIV ME on their rights and well-being. The Utility class focused on issues that could affect the scientific quality and credibility of HIV ME – issues related to principles of scientific integrity, transparency, and accountability, which are essential for ensuring the validity and reliability of research and public health interventions. The Utility class members may have a higher expectation and demand for HIV ME to demonstrate its value and utility for improving HIV prevention and care outcomes; they may be more skeptical of the benefits of HIV ME and more critical of the limitations and uncertainties of HIV ME.

It is critical to understand how stakeholders prioritize relevant ethical concerns to enable the weighing of benefits versus risks and related tasks of formulating and revising research and public health guidelines. By aligning new and updated guidelines with stakeholders’ priorities and preferences, we stand to enhance their effectiveness. For instance, insights from our study relate to the efforts undertaken by interdisciplinary working groups to improve the ethical conduct of HIV phylogenetic research [47]. These working groups identified critical issues such as study design, data security, access, and sharing; community engagement; and communication and dissemination. Studies such as ours can help recognize which of these issues matter most to stakeholders and can inform the decisions that address them. Similarly, insights from our study could further inform the CDC’s guidance for health departments conducting cluster detection and response, which the CDC updated in February 2024 [46]. The CDC indicated that its new guidance incorporates input from HIV and human rights organizations who sent a letter about their concerns with molecular HIV surveillance [48], including concerns considered by our study such as individual consent and opt-out. In summary, stakeholders’ priorities play a pivotal role in shaping policy decisions and the ethical landscape of HIV-related research.

This study presented some unique challenges. There is a small and finite number of researchers and PHPs involved in HIV ME. This is a particular issue with the U.S. public health workforce, especially in the context of the COVID-19-related burnout [49]. A recent study found that nearly half of all public health workers in state and local agencies left their jobs between 2017 and 2021, creating many vacancies [50]. Moreover, there are limited ways to identify PHPs who work in U.S. public health departments. These factors influenced our approach to recruitment. We carefully considered expanding the scope of our survey by inviting a broader range of participants. However, after thoughtful deliberation, we decided against it. Our concern was that doing so could introduce too much heterogeneity and bias the results to the null. Instead, we intentionally focused on a specific population of professionals who possessed the technical skills for conducting HIV ME or applying it in public health. Importantly, we recognize that this population is both small and finite. Our survey, however, posed questions about ethical issues that may not necessarily be apparent to all those with the technical skills to conduct or apply it. The professionals who participated in our survey therefore constitute a unique population. They not only possess the technical skills and knowledge to conduct or apply HIV ME, but also demonstrate the expertise to consider its broader context. By engaging this population, our survey is among the first to systematically and comprehensively capture the perspectives of professionals who can meaningfully contribute to discussions on ethical matters within this field.

We faced interesting questions in determining the appropriate survey framework. Initially, our focus was centered on the technologies used for HIV ME. However, through engaging with expert stakeholders we realized that this approach was too narrow in scope. Additionally, we deliberated on how to effectively present the ethical issues associated with HIV ME, enabling researchers and PHPs to thoroughly consider these issues. This was particularly important when the issues highlighted differences in existing rules, norms, and practices between the groups such as around disclosing use of information, obtaining individual consent, and providing opt-out options. For example, research use of HIV ME typically involves enrolling individuals into research studies via informed consent and provides opportunities to opt-out. In contrast, individual consent and opt-out is not typical in public health practice. Input from our expert stakeholders was critical for refining how these issues were described while supporting our intention to understand similarities and divergences in perspectives that could inform, and shape future discussions related to the policies and practices of HIV ME.

Another challenge related to the staged implementation of molecular HIV surveillance where public health departments have not implemented molecular HIV surveillance at the same time. For instance, some public health departments implemented it several years ago when the CDC first piloted the initiative and others are only beginning the implementation process. Therefore, PHPs have varied experiences with HIV ME depending on their public health jurisdiction. Our survey was not designed to account for these types of variations in PHPs’ experience.

When contemplating whether to compensate survey respondents, our team accounted for the fact that individuals employed at U.S. public health departments are ineligible to receive incentives for their participation. We recognized that compensating only some participants while excluding others based on their workplace would be inequitable. So, after thoughtful deliberation, we decided not to provide compensation to any of the participants. Ethical considerations surrounding participant compensation are of critical importance in research and survey studies. Offering compensation is a common practice to acknowledge the time and effort invested by participants and promote a fair exchange between researchers and the individuals contributing their valuable insights. While our decision excluded compensation for all participants, we had a decent response rate and believe the approach reduced any perceived favoritism or unequal treatment and reinforced the integrity of the study.

This study demonstrates the usefulness of BWS to systematically explore the ethical issues associated with the use of HIV ME in the United States. We found heterogeneity among respondents in their prioritization of ethical issues reflecting their different perspectives and priorities on the potential social harms and scientific utility of the data. Knowing these differences exist can shape future deliberations on policies and practices of HIV ME to address these ethical issues. Future studies should use BWS to examine the perspectives of additional stakeholders, including persons living with HIV and persons living without HIV at increased risk of acquiring it, to ensure their voices are represented in all discussions about the policies and practices of HIV ME.

### Supplementary Information

Supplementary Material 1.

## Data Availability

The datasets used and/or analyzed during the current study are available from the corresponding author on reasonable request.
