# Supplementary material for: Prioritization of ethical concerns regarding HIV molecular epidemiology by public health practitioners and researchers
Source: BMC Public Health. 2024 May 29;24:1436. doi: 10.1186/s12889-024-18881-4 (PMC11137925; doi:10.1186/s12889-024-18881-4)
Supplement: Supplementary file 1 — Supplementary Material 1. [file 12889_2024_18881_MOESM1_ESM.docx]

**Supplementary material: Best-worst scaling (BWS) instrument**

We are going to present 11 ethical issues that may arise when using HIV molecular epidemiology for research or public health activities in the United States. We want to understand the extent to which people in your profession are concerned about these ethical issues.

1. **Limited evidence of benefits**: HIV molecular epidemiology results may be used to inform HIV prevention and treatment strategies, and ultimately reduce HIV transmissions. The evidence may be limited regarding the benefits of using HIV molecular epidemiology. Evidence from controlled trials or prospective empirical observations could further establish its benefits.

Are people in your profession concerned that there is limited evidence of benefits related to HIV molecular epidemiology?

- Yes
- No

1. **Lack of individual consent**: Obtaining informed consent involves providing individuals with comprehensive information about HIV molecular epidemiology, including its purpose, risks, benefits and uses of data, and then obtaining the individual’s voluntary agreement to participate. Some programs may not be required to obtain individual consent for the use of data in HIV molecular epidemiology, such as for HIV surveillance or research involving de-identified data.

Are people in your profession concerned about a lack of consent to use data for HIV molecular epidemiology?

- Yes
- No

1. **Lack of directly disclosing data use**: Disclosure refers to informing individuals that their personal information, such as their HIV genetic sequences, will be used by programs for HIV molecular epidemiology prior to collecting it. Some programs do not routinely disclose the use of an individual’s information prior to it being collected.

Are people in your profession concerned about a lack of directly disclosing data use to individuals whose data are used in HIV molecular epidemiology?

- Yes
- No

1. **Lack of an opt-out option**: An opt-out option refers to a process where individuals can withdraw their data from use after it has been collected. Some programs that use HIV molecular epidemiology do not routinely give individuals an opt-out option.

Are people in your profession concerned about a lack of an opt-out option for individuals to withdraw their data from use in HIV molecular epidemiology?

- Yes
- No

1. **Limited resources for other activities**: HIV molecular epidemiology may be used to prioritize public health resources for activities expected to have a greater impact on increasing case detection and interrupting HIV transmission. However, it is unclear how the use of resources for HIV molecular epidemiology impacts the allocation of resources for other standard HIV public health activities.

Are people in your profession concerned that the use of resources for HIV molecular epidemiology limits resources for other activities?

- Yes
- No

1. **Infer source of HIV transmission**: The use of HIV molecular epidemiology may enable programs to identify persons they believe to be the source of a particular infection. This can be a concern though because there is always uncertainty in the results and that uncertainty may be lost when reporting or acting on the results.

Are people in your profession concerned about programs being able to infer the source of HIV transmission based on results from HIV molecular epidemiology?

- Yes
- No

1. **Infer directionality of HIV transmission**: Programs using HIV molecular epidemiology may be able to infer the directionality of HIV transmission (i.e., who transmitted HIV to whom). The ability to infer the directionality of HIV transmission depends on different factors including the methods used to generate the datasets. There are questions about the value of being able to infer the directionality of HIV transmission.

Are people in your profession concerned about programs being ability to infer the directionality of HIV transmission based on results from HIV molecular epidemiology?

- Yes
- No

1. **Increased risk of harm towards individuals**: Results from programs using HIV molecular epidemiology can potentially reveal characteristics about individuals that increase their risk of experiencing discrimination. Discrimination is often a consequence of stigma and can occur when unfair actions are taken against individuals based on their belonging to a stigmatized group.

Are people in your profession concerned that results from the use of HIV molecular epidemiology could increase the risk of harm towards individuals?

- Yes
- No

1. **Increased risk of stigma towards groups**: Results from programs using HIV molecular epidemiology could lead to increased stigma towards communities over-represented in the HIV epidemic. Communities disproportionately affected by HIV are also often affected by stigma associated with, among other things, gender identity, sexual behavior, or use of injection drugs.

Are people in your profession concerned that results from the use of HIV molecular epidemiology could increase the risk of stigma towards groups?

- Yes
- No

1. **Increased risk of legal prosecution**: In many jurisdictions of the United States, HIV transmission and nondisclosure of HIV status are criminal offenses. If results from HIV molecular epidemiology could be used to infer who transmitted HIV to whom, they might increase the risk of individuals being legally prosecuted.

Are people in your profession concerned that results from the use of HIV molecular epidemiology could increase the risk of legal prosecution?

- Yes
- No

1. **Re-use of data collected for clinical purpose**s: Some programs systematically re-use data collected during routine HIV clinical care for HIV molecular epidemiology. The re-use processes may not promote shared decision making about data usage between persons living with HIV and their clinician. They may also not promote trust between persons living with HIV, their clinician, and the programs re-using the data.

Are people in your profession concerned about the re-use of data that was collected for clinical purposes for HIV molecular epidemiology?

- Yes
- No

**____________________________________________________________________________**

We now want to understand the extent to which people in your profession are concerned about these 11 ethical issues. We will do so through a series of 11 questions.

Each question will show you a unique subset of the 11 ethical issues and ask you to select the one issue that is most concerning and then the one issue that is least concerning.

**____________________________________________________________________________**

Before we begin, we will show you an example of the questions we will ask.

We will say that a person named Alex is asked to select the one issue that is most concerning and then the one issue that is least concerning about the use of HIV molecular epidemiology for research or public health in the United States.

| Most concerning |  | Least concerning |
| --- | --- | --- |
|  | Increased risk of harm towards individuals |  |
|  | Lack of individual consent |  |
|  | Limited evidence of benefits |  |
|  | Limited resources for other activities |  |
|  | Increased risk of legal prosecution |  |

Alex’s answer means that Alex is most concerned about the increased risk of legal prosecution and least concerned about limited resources for other activities.

**____________________________________________________________________________**

Now it is your turn. We will ask you 11 questions following this same format.

**Task 1.**

| Please choose the one issue that is **most concerning** and then the one issue that is **least concerning** regarding the use of HIV ME for research or public health in the United States. | | |
| --- | --- | --- |
| Most concerning |  | Least concerning |
|  | Increased risk of harm towards individuals |  |
|  | Re-use of data collected for clinical purpose |  |
|  | Limited evidence of benefits |  |
|  | Limited resources for other activities |  |
|  | Increased risk of legal prosecution |  |

**Task 2.**

| Please choose the one issue that is **most concerning** and then the one issue that is **least concerning** regarding the use of HIV ME for research or public health in the United States. | | |
| --- | --- | --- |
| Most concerning |  | Least concerning |
|  | Infer directionality of HIV transmission |  |
|  | Increased risk of legal prosecution |  |
|  | Re-use of data collected for clinical purposes |  |
|  | Lack of an opt-out option |  |
|  | Increased risk of stigma towards groups |  |

**Task 3.**

| Please choose the one issue that is **most concerning** and then the one issue that is **least concerning** regarding the use of HIV ME for research or public health in the United States. | | |
| --- | --- | --- |
| Most concerning |  | Least concerning |
|  | Lack of an opt-out option |  |
|  | Infer directionality of HIV transmission |  |
|  | Lack of an opt-out option |  |
|  | Increase risk of harm towards individuals |  |
|  | Re-use of data collected for clinical purposes |  |

**Task 4.**

| Please choose the one issue that is **most concerning** and then the one issue that is **least concerning** regarding the use of HIV ME for research or public health in the United States. | | |
| --- | --- | --- |
| Most concerning |  | Least concerning |
|  | Lack of directly disclosing data use |  |
|  | Infer source of HIV transmission |  |
|  | Infer directionality of HIV transmission |  |
|  | Re-use of data collected for clinical purposes |  |
|  | Limited |  |

**Task 5.**

| Please choose the one issue that is **most concerning** and then the one issue that is **least concerning** regarding the use of HIV ME for research or public health in the United States. | | |
| --- | --- | --- |
| Most concerning |  | Least concerning |
|  | Limited evidence of benefits |  |
|  | Lack of an opt-out option |  |
|  | Limited resources for other activities |  |
|  | Increased risk of stigma towards groups |  |
|  | Lack of directly disclosing data use |  |

**Task 6.**

| Please choose the one issue that is **most concerning** and then the one issue that is **least concerning** regarding the use of HIV ME for research or public health in the United States. | | |
| --- | --- | --- |
| Most concerning |  | Least concerning |
|  | Lack of individual consent |  |
|  | Limited resources for other activities |  |
|  | Infer source of HIV transmission |  |
|  | Increased risk of legal prosecution |  |
|  | Lack of an opt-out option |  |

**Task 7.**

| Please choose the one issue that is **most concerning** and then the one issue that is **least concerning** regarding the use of HIV ME for research or public health in the United States. | | |
| --- | --- | --- |
| Most concerning |  | Least concerning |
|  | Limited resources for other activities |  |
|  | Increased risk of harm towards individuals |  |
|  | Increased risk of stigma towards groups |  |
|  | Lack of individual consent |  |
|  | Infer directionality of HIV transmission |  |

**Task 8.**

| Please choose the one issue that is **most concerning** and then the one issue that is **least concerning** regarding the use of HIV ME for research or public health in the United States. | | |
| --- | --- | --- |
| Most concerning |  | Least concerning |
|  | Infer source of HIV transmission |  |
|  | Increased risk of stigma towards groups |  |
|  | Increased risk of legal prosecution |  |
|  | Lack of directly disclosing data use |  |
|  | Increased risk of harm towards individuals |  |

**Task 9.**

| Please choose the one issue that is **most concerning** and then the one issue that is **least concerning** regarding the use of HIV ME for research or public health in the United States. | | |
| --- | --- | --- |
| Most concerning |  | Least concerning |
|  | Increased risk of stigma towards groups |  |
|  | Limited evidence of benefits |  |
|  | Lack of individual consent |  |
|  | Infer source of HIV transmission |  |
|  | Re-use of data collected for clinical purposes |  |

**Task 10.**

| Please choose the one issue that is **most concerning** and then the one issue that is **least concerning** regarding the use of HIV ME for research or public health in the United States. | | |
| --- | --- | --- |
| Most concerning |  | Least concerning |
|  | Re-use of data collected for clinical purposes |  |
|  | Lack of directly disclosing data use |  |
|  | Lack of an opt-out option |  |
|  | Increase risk of harm towards individuals |  |
|  | Lack of individual consent |  |

**Task 11.**

| Please choose the one issue that is **most concerning** and then the one issue that is **least concerning** regarding the use of HIV ME for research or public health in the United States. | | |
| --- | --- | --- |
| Most concerning |  | Least concerning |
|  | Increased risk of legal prosecution |  |
|  | Lack of individual consent |  |
|  | Lack of directly disclosing data use |  |
|  | Infer directionality of HIV transmission |  |
|  | Limited evidence of benefits |  |
